# Evaluation of the Effect of Binary Fly Ash-Lime Mixture on the Bearing Capacity of Natural Soils: A Comparison with Two Conventional Stabilizers Lime and Portland Cement

**DOI:** 10.3390/ma16113996

**Published:** 2023-05-26

**Authors:** Yhan P. Arias-Jaramillo, Diana Gómez-Cano, Gloria I. Carvajal, César A. Hidalgo, Fredy Muñoz

**Affiliations:** 1 Department of Construction, School of Architecture, Universidad Nacional de Colombia, Medellín 050034, Colombia; digomezca@unal.edu.co; 2 Engineering Faculty, Universidad de Medellín, Medellín 050026, Colombia; gicarvajal@udem.edu.co (G.I.C.); chidalgo@udem.edu.co (C.A.H.); 3 Engineering Faculty, Universidad Cooperativa de Colombia, Medellín 050012, Colombia; fredy.munozc@udem.edu.co

**Keywords:** soil stabilized, slimy soil, sandy soil, clayey soil, fly ash, pozzolanic effect, mechanical behavior

## Abstract

This study evaluates a binary mixture of fly ash and lime as a stabilizer for natural soils. A comparative analysis was performed on the effect on the bearing capacity of silty, sandy and clayey soils after the addition of lime and ordinary Portland cement as conventional stabilizers, and a non-conventional product of a binary mixture of fly ash and Ca(OH)_2_ called FLM. Laboratory tests were carried out to evaluate the effect of additions on the bearing capacity of stabilized soils by unconfined compressive strength (UCS). In addition, a mineralogical analysis to validate the presence of cementitious phases due to chemical reactions with FLM was performed. The highest UCS values were found in the soils that required the highest water demand for compaction. Thus, the silty soil added with FLM reached 10 MPa after 28 days of curing, which was in agreement with the analysis of the FLM pastes, where soil moistures higher than 20% showed the best mechanical characteristics. Furthermore, a 120 m long track was built with stabilized soil to evaluate its structural behavior for 10 months. An increase of 200% in the resilient modulus of the FLM-stabilized soils was identified, and a decrease of up to 50% in the roughness index of the FLM, lime (L) and Ordinary Portland Cement (OPC)-stabilized soils compared to the soil without addition, resulting in more functional surfaces.

## 1. Introduction

The soil stabilization technique has been used in civil engineering applications, such as road construction, foundation and embankment, with the main objective of improving the microstructural behavior and mechanical performance of natural soils [1,2,3,4]. The bearing capacity of soil can be improved by physical treatments, such as particle size distribution [5,6,7], and chemical treatments through the reaction of soil components with a stabilizing material, traditionally lime or Portland cement [8,9,10,11]. However, highly alkaline industrial wastes can produce cementitious properties similar to those of Portland cement, exhibiting the potential to partially or even totally replace conventional materials [12,13,14,15]. Previous studies confirmed the feasibility of using fly ash from coal combustion in industrial boilers [16,17] as a cementitious material, due to its reaction capacity in the production of geopolymers, pastes and mortars [18,19], and soil stabilization applications [20,21].

The addition of fly ash at levels up to 24% can reduce sulfates in soils, reducing swelling of low plasticity clay soils of up to 1.5% (acceptable levels of <5.0%) [10]. Additionally, it has been reported that sulfate content greater than 0.25% by soil mass could generate expansion processes due to the formation of ettringite on contact with lime, which causes soil fracturing over time [22]. Mccarthy et al. have studied the addition of fly ash with high calcium content (type C), finding calcium silicate hydrate (CSH) formation, characteristic of the tobermorite phase, and an increase in soil cohesion and, therefore, in its elastic modulus [23]. It is corroborated that tobermorite is formed by pozzolanic reactions of fly ash, but also when aluminates and silicates of soil come into contact with calcium oxide.

Research studies have investigated the mechanical behavior of soil with the purpose of improving its performance and durability, but also for reducing its cost and environmental impact [24,25,26,27,28]. Current findings have indicated the optimal conditions for stabilizing soils, while considering the percentage of addition (waste) and its physicochemical and mineralogical characteristics [29,30,31]. These studies have also demonstrated that parameters such as optimum moisture and maximum dry weight are inversely affected by the percentage of addition due to an increase in the specific surface area. Additionally, studies have found increases of up to 600% in the unconfined compressive strength of soils with fly ash additions between 10% and 14% of the soil mass, compared to soils with no additions [32,33,34,35].

The aim of this study was to evaluate the mechanical and microstructural behavior of soils (silty, sandy and clayey) added with a mixture of fly ash and lime (FLM), as an alternative material for soil stabilization, compared to conventional stabilizers such as OPC and Lime (L). In this sense, due to the hydraulic nature of the binary mixture of lime and fly ash (FLM), it is necessary to know the cementitious behavior and the physical–mechanical response to the water demand of the soil in order to achieve optimum soil compaction moisture. 

Finally, it is noted that reproducing the laboratory behavior on a test track, stabilizing a soil belonging to an unpaved road, allows not only the verification of the behavior of the FML with respect to a non-stabilized soil, but also the comparison with traditional stabilizers such as OPC and Lime (L). This makes it possible to establish new stabilization alternatives that are economical, technically functional, and with an important environmental component [36].

## 2. Materials and Methods

### 2.1. Materials and Characterization

Three native soil types were used, namely silt (M), sand (S), and clay (C), from La Unión and Medellín, Antioquia, Colombia. The physical characteristics of the soils studied are shown in Table 1, while Table 2 shows their chemical composition determined by X-ray fluorescence (XRF), using a Phillips PW 2400 X-ray fluorescence spectrometer.

Ordinary Portland cement (OPC, general use) was used, with an average particle size of less than 45 μm, and commercial lime (L, type N) of which 95% passed through the 325 sieve. A binary mixture of fly ash and lime (FLM) was used as the alternative material, with a proportion of 75% and 25%, respectively. The fly ash was collected from coal combustion in a thermoelectric plant used for the energy supply of a regional textile company in Itagüi, Antioquia, Colombia. Fly ash has a particle size of less than 75 μm. Table 3 shows the chemical composition of fly ash and lime.

**Figure 1:**
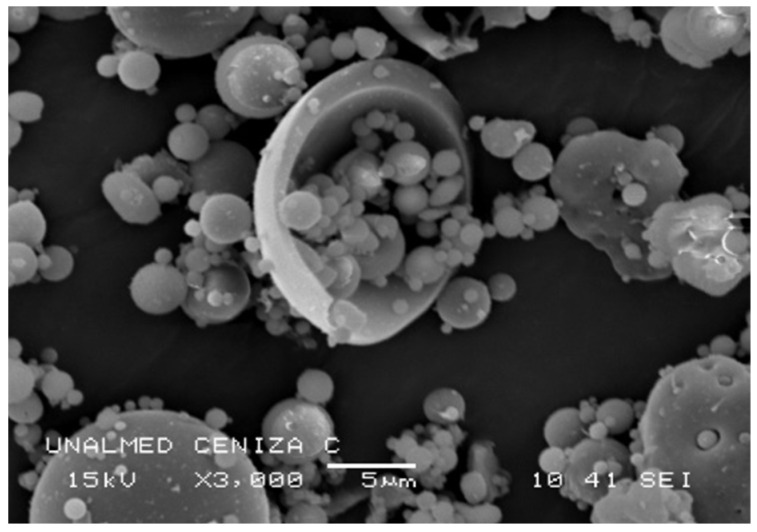
Scanning electron microscopy (SEM) images of fly ash.

Figure 1 shows the morphology of the fly ash particles taken by scanning electron microscopy (SEM), with a JEOL JSM 5910LV SEM with 15 kV back-projected electron detectors and 10 mm working distance, where a hollow spherical shape (exosphere type) is evident, which is a typical characteristic of reactive fly ash due to its large surface area.

**Figure 2:**
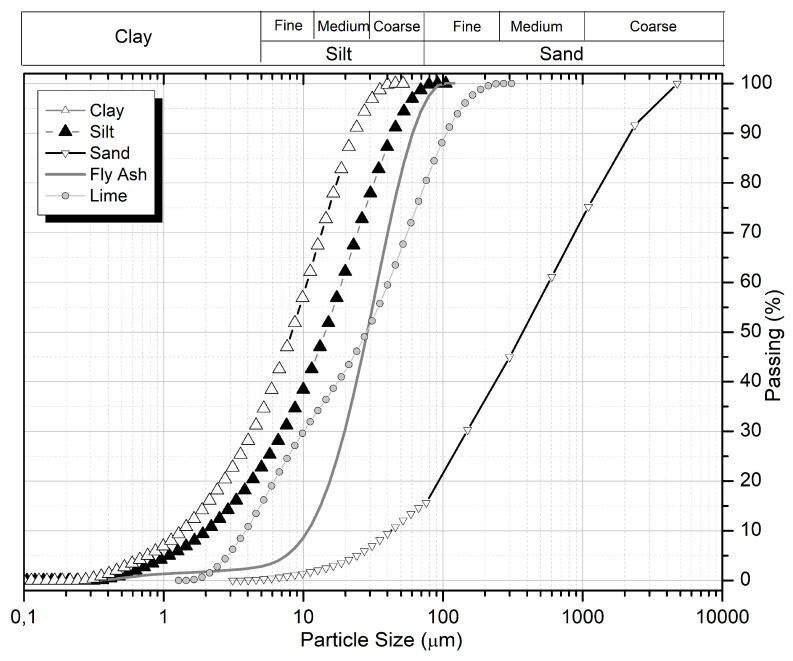
Particle size distribution of soils.

Figure 2 shows the particle size distribution of soils and fly ash (ASTM D6913-17), indicating the relationship between the average particle size and the specific surface area of fine soils and fly ash. 

Table 4 presents the characteristic size distributions D_10_ (10% pass), D_50_ (50% pass), and D_90_ (90% pass) and the specific area using a Master Sizer 3000 equipment. 

The mineralogical analysis of the fly ash and soils was performed by X-ray diffraction (XRD) on a PANa-lytical X’Pert MPD PRO between 5° and 60° for 120 min with CuKα1 source (λ = 1.54059 Å). The findings are presented in the results section. 

### 2.2. Methods

#### 2.2.1. Experimental Development in Pastes 

Pastes of the binary mixture (FLM) and water were prepared to evaluate the binder capacity. FLM pastes were manufactured using 10%, 20%, and 40% of their dry weight in water, which were equivalent to a water/binder ratio of 0.10, 0.20, and 0.4, respectively. The pastes were poured into polyvinyl chloride (PVC) molds with an aspect ratio of 1:2 (D:25 mm) and cured at 40 °C in plastic containers. 

Uniaxial compressive strength (UCS) was determined as a response variable at 7, 28 and 56 days of paste curing, using a Humboldt 3000 machine with a load cell of 45 kN at a speed of 0.5 mm/min). Curing times were selected in order to study the pozzolanic activity of FLM in both the short and long term. Finally, the chemical activity of FLM was evaluated for 20% moisture pastes at 7, 28, and 56 days, and 10% and 40% moisture pastes at 56 days by mineralogical analysis using X-Ray Diffraction (XRD).

#### 2.2.2. Experimental Development in Stabilized Soils

- Maximum dry weight and optimum moisture

Twenty one modified proctor compaction curves were performed to determine the maximum dry weight and optimum moisture content of each soil mixture, added with 7% and 24% FLM, OPC, and L. Table 5 shows the compaction test performed according to ASTM D1557-12.

**Figure 3:**
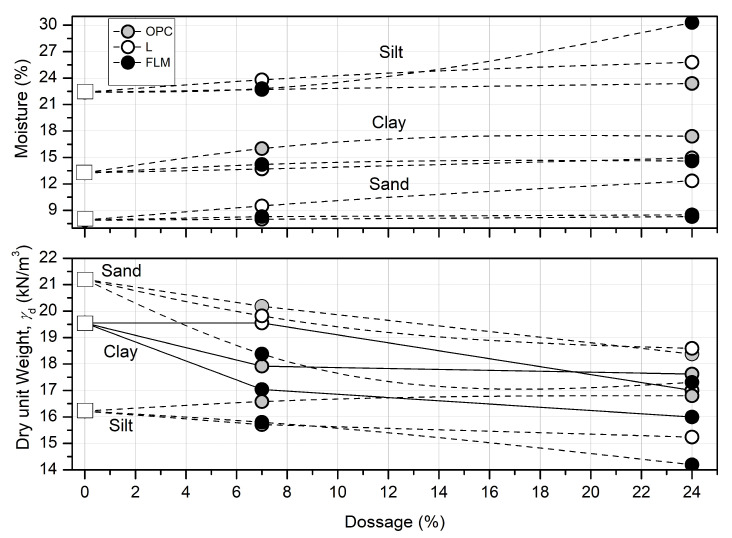
Proctor curves.

Figure 3 shows the variation of optimum moisture and maximum dry unit weight, respectively. Compaction curves were constructed according to the information in Table 5, to determine the optimum moisture content and maximum dry weight of the soils. The soil mixtures (M, S and C) were designed with 0%, 3%, 6%, 9%, 12% and 24% by mass of FLM, OPC and L, respectively.

- Bearing capacity of soils and mixtures

To determine the compressive strength of the stabilized soils, cylindrical specimens of 50 mm in diameter and 100 mm in length were manufactured. The specimens were packaged in plastic containers and cured at 40 °C until reaching a curing age of 7, 28 and 56 days. Before testing, a drying process was performed to obtain a moisture content of 5% ± 2% in each specimen. Four specimens were prepared for each combination, corresponding to a total of 216 specimens, including all combinations of dosage and curing times. In this case, a Humboldt 3000 machine was used with a 45 kN load cell and a speed of 0.5 mm/min. 

In order to demonstrate the effect of FLM addition on soils, a mineralogical analysis using XRD was performed on each soil mixture (M, S, and C) with added 14% FLM at a curing age of 56 days.

Finally, a granulometric analysis of the added soils was carried out to determine the best adjustment of soil particles, which would ensure physical densification. The Füller’s model defines a theoretical curve with a parabolic shape that approximates the maximum density and minimum void content gradation, as shown in the following equation:

(1) $$p=100\sqrt{\frac{d}{D}}$$

*p*: percentage by weight that passes through the sieve 

*d*: diameter of each sieve

*D*: maximum size

#### 2.2.3. Field Work

**Figure 4:**
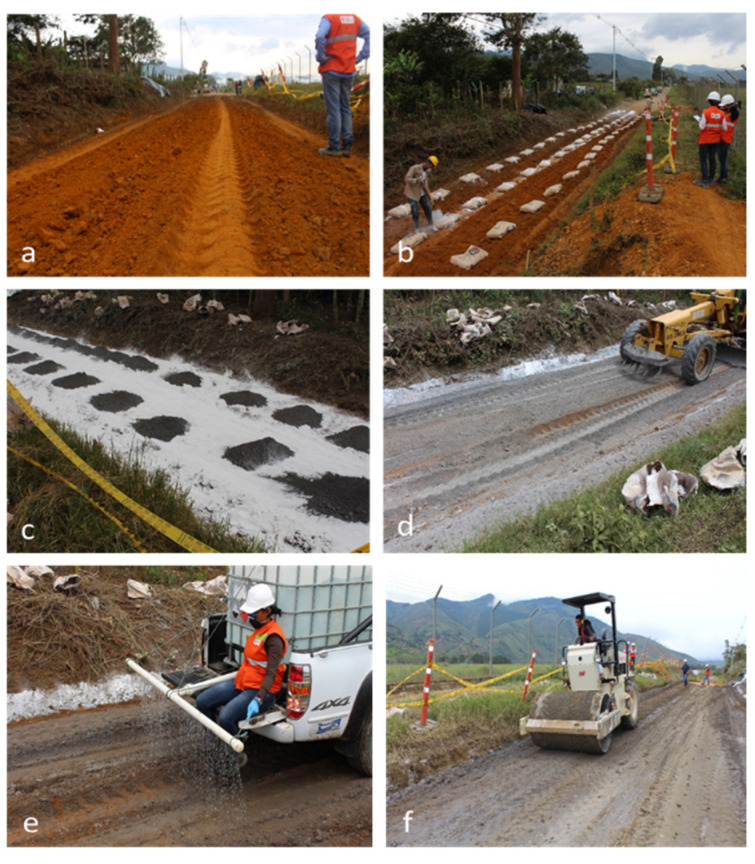
(**a**–**f**) Test track construction process.

To evaluate the strength and durability performance of a stabilized soil, a 120 m long test track was constructed, divided into four cells of 30 m. The test track was constructed on an unpaved road with low traffic volume, located in western Colombia (N06°19′41.4″, W076°08′24.3″), as shown in Figure 4.

The stabilized soil corresponded to the MH classification (see Table 1). All cells were constructed with a thickness of 0.2 m. The first cell was constructed with FLM-stabilized soil, the second with OPC, the third with hydrated lime (L), and the fourth was constructed without any additions to be used as a reference. The above were defined taking into account what previous research had shown [37,38,39].

The construction process of each cell is described as follows: (a) scarification and disintegration of the soil, (b) placement of the stabilizing material along the cell, according to the dosage obtained in the laboratory, (c) opening of the container containing the stabilizer, (d) homogenization of the soil with the stabilizing material, (e) wetting of the mixture, and (f) compaction. Subsequently, the compacted surface was cured by periodically wetting it and covering it with a polyethylene cover.

Finally, the durability and strength of the test track were carried out in service and taking into account traffic and weather conditions. The International Roughness Index (IRI) value was made, using the Transport and Highway Research Laboratory (TRL) Merlin low-cost road roughness measuring machine, and the resistance modulus using a low-load deflectometer (LWD), based on ASTM E2583. Both tests were performed on stabilized soil cells, and their evolution was noted over a period of 10 months. The process consisted of measuring the specific areas in six zones of each cell, specifically where tracks produced by tires were located in the left and right footprint at 10, 15 and 20 m from the beginning of the cell. No readings were obtained at the beginning and end, to avoid readings in transition zones with other stabilized cells.

## 3. Results

### 3.1. Development of Pastes

**Figure 5:**
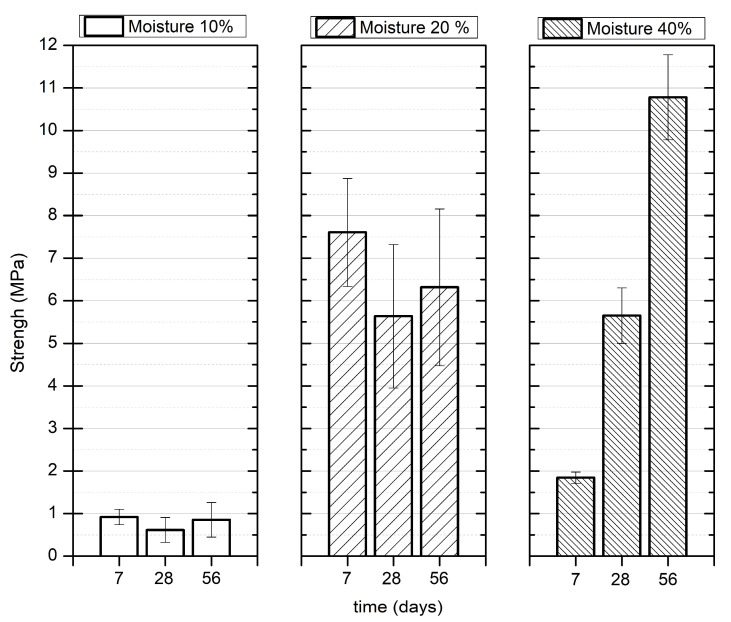
FLM pastes: effect of moisture on UCS.

Figure 5 shows the mechanical behavior of FLM pastes. It shows an increase in mechanical resistance in relation to the age of curing, mainly due to the pozzolanic reaction in the fly ash–lime system.

Pastes with 20% water exhibited the best mechanical response at seven days, indicating that this was an adequate performance condition for early ages. This could in turn be attributed to the high compaction achieved (12.65 kN/m^3^), which was 19% and 16% higher than the pastes with 10% (10.17 kN/m^3^) and 40% (10.55 kN/m^3^) moisture, respectively. In contrast, the low resistance observed in early curing in pastes with 10% moisture was due to the lower ionic mobility that decreased the reaction kinetics during the formation of phases with cementitious characteristics.

Finally, in the 40% moisture pastes, low mechanical response was associated with high porosity and low density reached at early ages. On the contrary, the excess water allowed a better reactivity at later ages, which exceeded that of the 20% moisture paste. Consequently, an 80% increase at 56 days of curing was obtained compared to the paste with 20% moisture at the same age.

**Figure 6:**
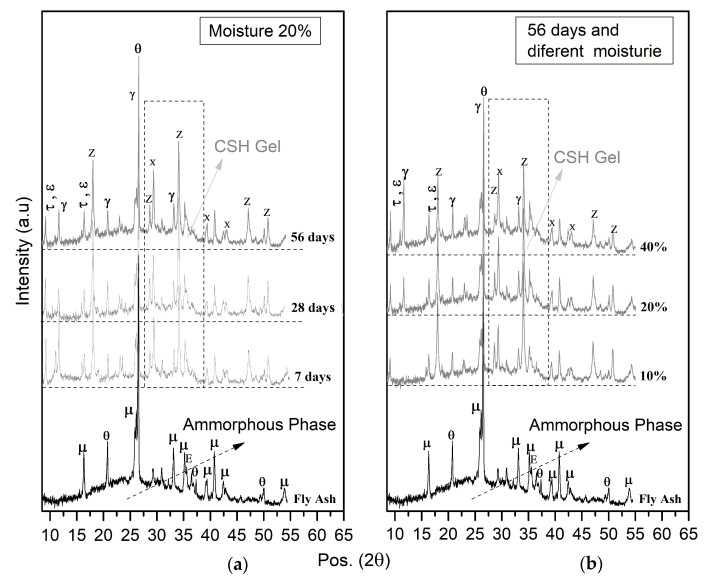
Mineralogical phases for FLM pastes showing the effect of (**a**) time and (**b**) moisture (μ: mullite, θ: quartz, E: hematita, τ: thaumasite, ε: ettringite, γ: gismondine, x: calcium carbonate, Z: calcium hydroxide).

The mineralogical analysis of FLM pastes is presented in Figure 6.

The presence of gismondine and the displacement of the amorphous halo in the XRD results is shown in Figure 6, which indicated pozzolanic activity and CSH formation in the evaluated (FLM) pastes that is strongly linked to the availability of water in the system. In contrast, mechanical responses were affected by the hybrid effect of the formation of CSH-type compounds and calcium carbonates CaCO_3_ (shown in XRD), and as reported by Kolias et al. [4].

### 3.2. Development of Stabilized Soils

**Figure 7:**
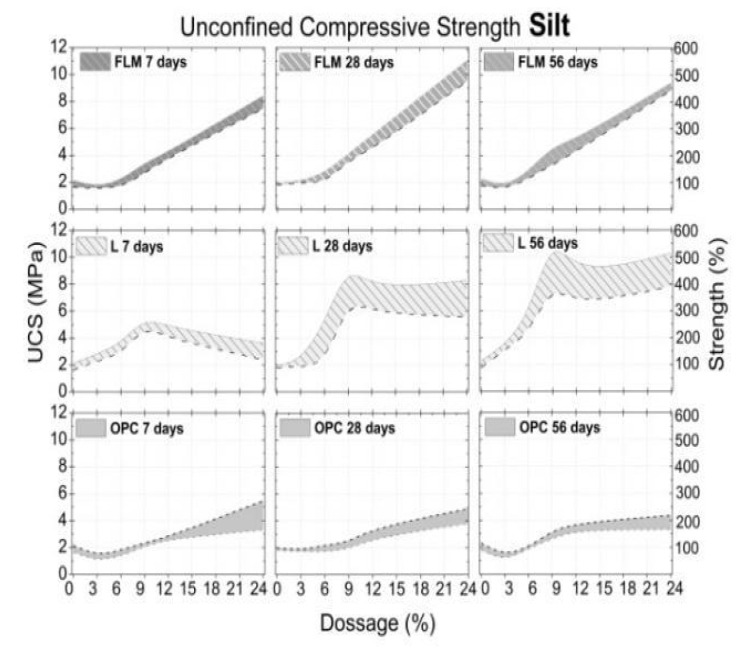
UCS of silt soil on different days of curing.

**Figure 8:**
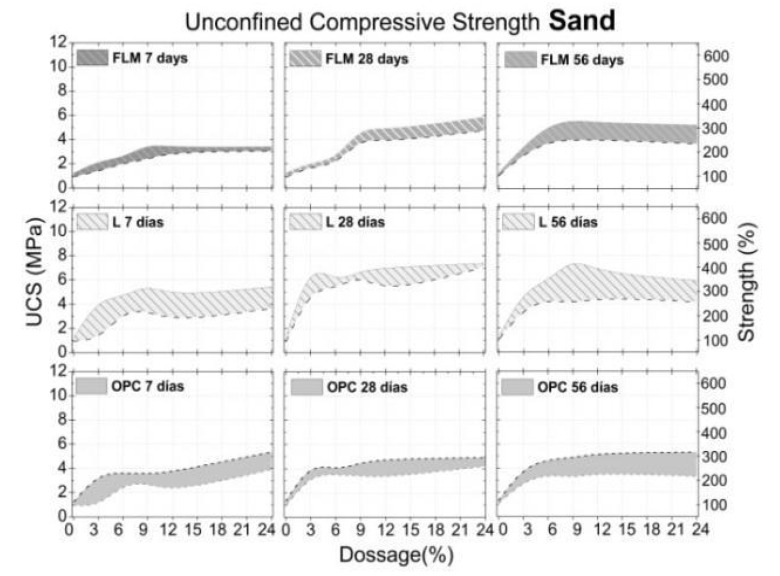
Sand-UCS on different days of curing.

**Figure 9:**
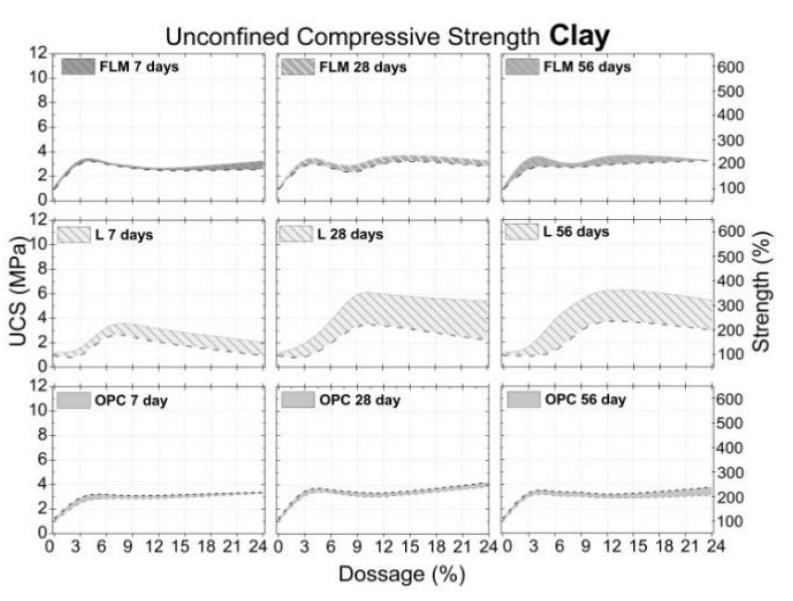
Clay-UCS on different days of curing.

The effect of adding FLM, OPC, and L in the UCS of soils (silt, sand and clay) is presented in Figure 7, Figure 8 and Figure 9, respectively. All graphs were constructed by assuming ± standard deviation values of the UCS mean (corresponding to four test specimens for each dosage and curing age). The confidence level was 67.5%.

As previously shown (Figure 3), addition of FLM, OPC, and L to silt soil (M) resulted in a moisture content between 21.5% and 30%. Figure 7 shows that the UCS of S soil was approximately 2 MPa with a standard deviation of 0.24 MPa. In general, the compressive strength of M soil increased with increasing FLM content, achieving a maximum of 10 MPa (450% increase) following the addition of 24% FLM. Addition of OPC to the UCS of M soil exhibited an asymptotic behavior after 12%. The maximum UCS achieved was 5 MPa (150% increase over the unstabilized soil). Finally, a curvature effect in the UCS was noted following the addition of L (maximum of 9%) to the M soil. In addition, we observed an increase in soil bearing capacity with an increasing age of curing. A maximum of 10 MPa (450% increase over the unstabilized soil) was observed at 56 days of curing. Both FLM and L systems comprised the optimal stabilization conditions for M soil.

Furthermore, as shown in Figure 3, addition of FLM, OPC, and L to sand soil (S) resulted in a moisture content between 7.5% and 12%. Figure 8 shows that the UCS values at different ages for this soil were approximately 1.16 Mpa with a standard deviation of 0.16. Addition of both FLM and OPC to S soil exhibited similar patterns over time, achieving a maximum UCS value of 5 Mpa with 6% of stabilizers (200% increase over the unstabilized soil). Addition of 9% L demonstrated the best performance, achieving UCS values of 7 Mpa (300% increase over the unstabilized soil).

**Figure 10:**
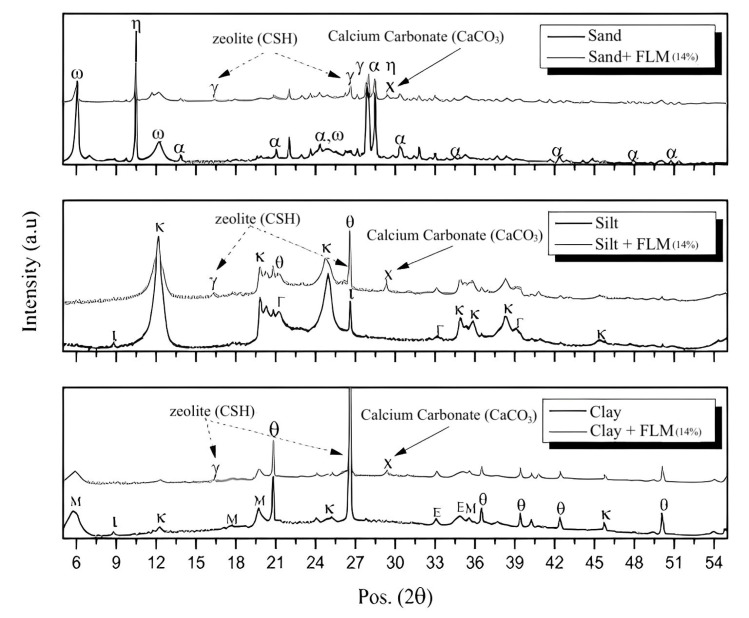
Mineralogical phases for soils: M: montmorillonite, θ: quartz, x: calcium carbonate, ε: ettringite, μ: mullite, ω: vermiculite, α: albite, η: hastirgsite, ι: illite, K: kaolinite, Γ: goethite.

Finally, the addition of FLM, OPC, and L to clay soil exhibited a moisture content between 13% and 18% (Figure 3). As shown in Figure 9, the UCS values for C soil were around 1.16 MPa with a standard deviation of 0.16. Addition of both FLM and OPC to C soil demonstrated a very similar behavior over time, achieving a maximum UCS value of 4 MPa when incorporating percentages of stabilizers higher than 3% (150% increase over the unstabilized soil). Addition of 12% L presented the best performance, achieving UCS values of 7 MPa (200% increase over unstabilized soil). Figure 10 shows the obtained XRD data at 56 days of curing for soils with added FLM at 14% by mass.

The sandy soil showed albite crystals associated with plagioclases and other constituents as micas, which were related to the presence of vermiculite. With the addition of FLM, a diminished presence of gismondine and calcium carbonate was observed. In contrast, the silty soil was characterized by the presence of quartz, kaolinite, and goethite, and exhibited characteristics of low plasticity, as shown in Table 1. The addition of FLM showed well-defined peaks of gismondine and calcium carbonate. 

Finally, the clayey soil contained quartz and montmorillonite as clay with a high cation exchange capacity (see Table 1), which is in agreement with [40]. Chemical and mechanical stabilization was implied due to the expansive and thixotropic character of clay. Incorporation of FLM revealed low peaks of gismondine and an intense presence of calcium carbonate. In summary, the results of all three soil types highlighted the presence of zeolite crystals, which is a distinct characteristic of the formation of gismondine-type phases, confirming the chemical activity of FLM as a stabilizer. 

**Figure 11:**
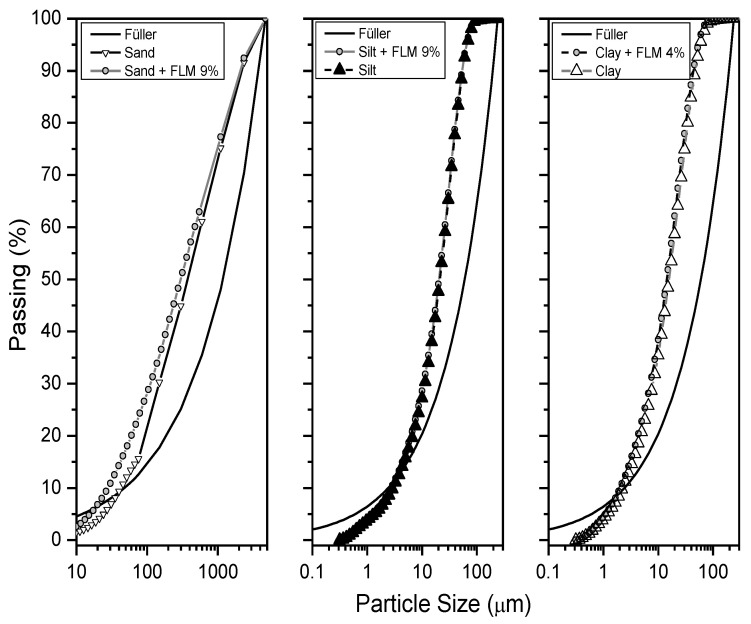
Fuller analysis.

Figure 11 shows variations in the particle size distribution of each soil, based on the added FLM. The percentage of FLM added to each soil represents the amount needed to achieve a 200% increase in soil bearing capacity. In addition, the Füller’s line reflects the distribution of particles associated with a suitable condition of physical densification.

The effect of adding fines was noticeable in sandy soil, as the new distribution (sand + FLM) diverges from the Füller curve. This indicates that the increased bearing capacity is associated with a chemical cementing effect and not with a physical size distribution effect. However, when compaction energy is applied, a new particle size distribution and plastic deformation is generated, contributing to the physical densification of the stabilized soil. Fine-grained soils showed no effect on granulometry, although there was a slight tendency to obtain a better fit in silty soil.

### 3.3. Field Work

**Figure 12:**
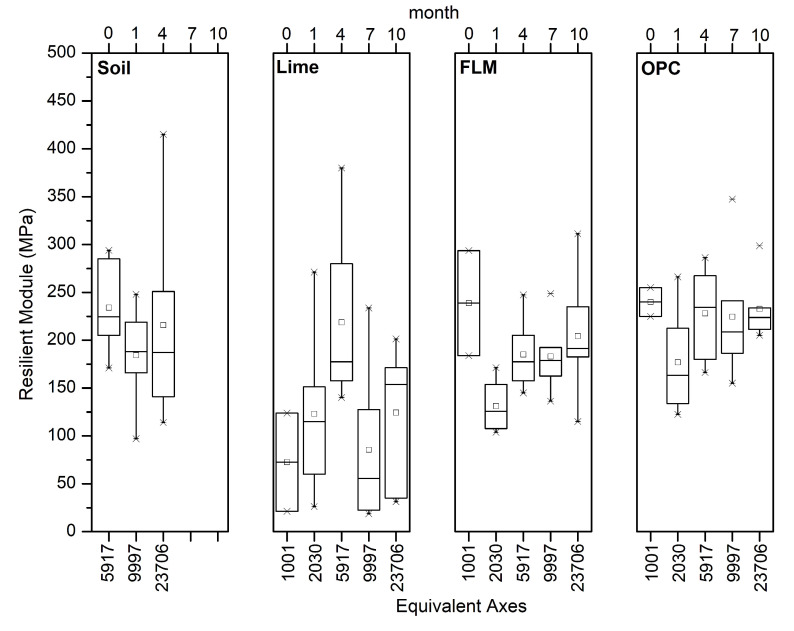
International Roughness Index (IRI) of stabilized soil.

Figure 12 shows the evolution of the International Roughness Index (IRI) with respect to the transit intensity represented as the number of 7.2-ton equivalent axes for each of the cells during the time of study. Additionally, an IRI criteria for road surfaces acceptation is presented established by the National Road Institute of Colombia-INVIAS. In general, these criteria have established four categories, namely very good, good, acceptable, and bad. It can be observed that the IRI values increased in each cell with passing time, indicating a deterioration in the conditions of cells due to climate and traffic stresses. In such cases, where the surface remains exposed, their combined effect induces erosion. However, it was observed that soil stabilization diminished the IRI progress compared with the unstabilized soil. 

Cells stabilized with FLM and cement exhibited the lowest IRI values at the end of the study, corresponding to acceptable conditions. The IRI values of the cell stabilized with L were also within the acceptable range despite being higher (sloping area), which increased the probability of soil erosion.

**Figure 13:**
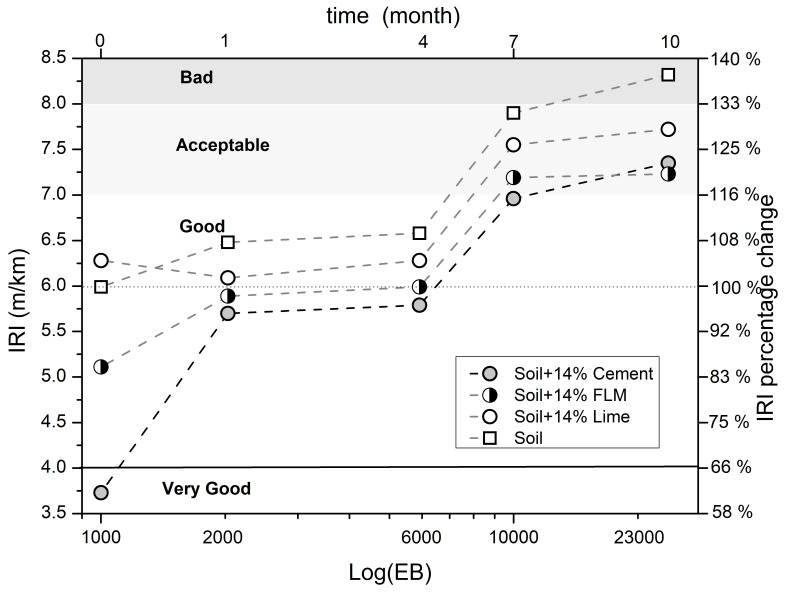
Resilient modulus of stabilized soil.

The evolution of the resilient modulus is shown in Figure 13, presenting its values at 4, 7, and 10 months. The cells stabilized with FLM and OPC showed a similar tendency to increase with time. Dispersion was remarkable in the results, possibly because of the excess cementitious material in some areas that might have accumulated during the construction process.

Figure 13 presents the measurements of the resilient modulus in boxplots showing the dispersion of the obtained data, and the variation associated with the construction process, possibly due to nonhomogenization in the test track. For both OPC and L, the results remained constant up to 10 months, guaranteeing continuity in the deformation capacity of the soil. In the case of FLM, an increase in the resilient modulus was noted, which might be associated with an improvement due to soil compaction with the equivalent axes and formation of cementitious compounds from the pozzolanic reaction, as shown in Figure 10. Similar values of resilient modulus were found for both FLM and L.

## 4. Discussion

The nature of the hydraulic binders OPC, L, and FLM, could explain the higher water demand for achieving maximum dry weight when added to each soil, which was higher in the case of silt, clay, and sand. Dissonantly, the density was affected by the specific gravity of stabilizers, being lower in the case of FLM addition followed by L. In OPC (specific gravity of 3.15), the dry weight increased with the additions in all three soils evaluated. Considering that it is necessary to cover the soil particles for the physicochemical effect of hydraulic binders to occur, the specific surface area of OPC (0.28 m^2^/g) was approximately 26% lower compared with fly ash and L, explaining the high demand of OPC for improving the bearing capacity in all three soil typologies. Addition of lime (L) to soils with a higher plasticity index (clay 17.30% and silt 21.00%) demonstrates a curvature effect reaching its maximum value after seven days of curing.

The UCS presented in the three soils with added FLM was associated with a hybrid effect between the primary source (fly ash) and the activator (lime). First, the physicochemical effect of calcium ions provided by lime, facilitated a decrease in the clay plasticity of soils (silt and clay). Second, the filling effect of the ash particles could achieve densification of the system by means of granulometric adjustment. Additionally, the pozzolanic effect in the soil–lime–ash interaction is not only present, but is also favored over time. In the cases of silt with a maximum UCS of 10MPa at 28 days, clay of 4 MPa independent of curing ages, and sand of 4.8 MPa, XRD confirmed their association with CaCO_3_ formation over time.

The highest UCS values were found in soils requiring the highest water demand for compaction (Figure 3). This is in agreement with the analysis of the FLM pastes, where soil moistures above 20% demonstrated the best mechanical characteristics (Figure 5). Therefore, these findings reveal a direct relationship with the improvement of ash reaction conditions, favoring the formation of CSH phases, as observed in the mineralogical analysis (Figure 6). Additionally, the pozzolanic effect of FLM on the slope of UCS as a function of the percentage of addition for silt indicates a strong relationship between improvements in soil properties and the amount added. For all trials, the best mechanical performance was provided with the use of lime (L) as a soil stabilizer, demonstrating a continuous improvement over time, mainly due to the formation of calcium carbonates (CaCO_3_) that chemically densified the matrix [10].

In all soils it was possible to observed that part of the lime associated with the FLM mixture reacted to form CSH phases, while another portion reacted to form calcium carbonates, confirming that complete reactivity of the binary mixture could not be guaranteed. However, both phenomena were found to be associated with an improvement in the bearing capacity of the soil. For OPC as a stabilizing material, the best performance was found in the sandy granular soil, which was 50% higher than that in silty and clayey soils. There was also no increase in UCS with curing time, thus the properties of all soils were maintained after seven days of curing.

The resilient modulus of FLM demonstrated values comparable with OPC and L. This phenomenon could be explained by pozzolanic reactions with the stabilizing materials; however, its value tended to decrease with time due to a combination of climatic conditions and traffic loads, indicating the presence of mechanical deterioration on the stabilized layer. Figure 13 shows that the sections stabilized with FLM and OPC exhibited a tendency to increase over time (resilient modulus up to 165 MPa and 175 MPa, respectively). In addition, the section stabilized with FLM continued to increase over time with respect to the section stabilized with OPC, presenting itself as an alternative material with high potential for soil stabilization. The section stabilized with FLM presented higher values, increasing its resistance value by up to 200%. 

The international roughness index (IRI) showed a better behavior over time. That is, when the soil was stabilized with FLM, OPC and L, “very good” IRI values were identified at the beginning of the tests, and “acceptable” values at the end of the period (see Figure 12). This behavior is normal due to the combined action of traffic and climate, where the surface is subjected to loading and wear conditions because the soil is fully exposed. On the other hand, the non-stabilized soil presented unfavorable conditions at the end of the tests, indicating that stabilized soils can guarantee greater durability.

Furthermore, although it was noted that the test track stabilized with OPC presented a better behavior over time, the soil stabilized with FLM reached a similar behavior after 2000 equivalent axes (differences between both stabilizers of less than 5% in the IRI value), see Figure 13. The progressive increase in the resistance of the soil stabilized with FLM (Figure 13), produced a harder wearing layer and greater resistance to wear caused by erosive processes; consequently, a greater homogeneity in the whole area was achieved. In this sense, the use of FLM could present behaviors compatible with cement.

## 5. Conclusions

In general, the three soils (M, S and C) added with an alternative stabilizer (FLM) demonstrated increased bearing capacity. However, soils requiring high water demands (approximately 20%) to achieve maximum compaction, will be most efficient when stabilized with FLM.

To ensure greater ion mobility and improve the reaction kinetics between ash and lime in a stabilizer such as FLM, it is important to implement effective curing processes by adding water to the soil once compacted, thus achieving a significant increase in soil grain cohesion, which is reflected by the improved bearing capacity and durability. Resistance to climatic agents and vehicular traffic is one of the most essential factors to consider in road improvement processes. The resilient modulus is a significant indicator that allows us to identify the degree to which the road has deteriorated, which increases as a result of pozzolanic reactions with the stabilizing materials.

The use of stabilizing materials improves the mechanical and functional conditions of roads in unpaved conditions, achieving more uniform surfaces that are more resistant to traffic loads and climatic events. However, it is necessary for the laboratory conditions to be reproduced at the site, applying a rigorous curing process, through constant moistening and protection, which allows an adequate pozzolanic reaction that guarantees greater resistance and durability from the moment it is put into service.

The FLM can be considered as an alternative for soil stabilization in low traffic volume roads. This is due to its high performance in the resistance obtained in comparison with non-stabilized soils (increase of up to 200%) and its tendency to increase over time despite the accumulation of rainfall events and traffic loads that trigger erosive processes. It also shows good functional behavior in its surface layer, with values between “good” and “acceptable”. Since it presents similar values to soils stabilized with OPC and L, and since it is composed of residual materials from the industry, the FLM is a potential stabilizer for the improvement of unpaved roads and even for the improvement of pavement structure grades and subgrades.

Finally, it is recommended that in future stabilizations, a constant comparison of the resilient modulus with the hydrological behavior of the area be carried out, which will allow the delimitation of differences such as those obtained at the beginning of the tests and improvement of the interpretation processes. This is due to the fact that the measuring equipment (lightweight deflectometer) is linked to soil moisture.

## Figures and Tables

**Table 1:** Geotechnical characteristics of natural soils.

Properties	Value		
	Silt	Sand	Clay
^1^ Liquid limit-LL (%)	55	-	41
^1^ Plastic limit-PL (%)	34	-	23.70
^1^ Plasticity index-PI (%)	21	-	17.30
^2^ Specific gravity		2.72	
^2^ Dry unit weight (kN/m^3^)	16.22	21.20	19.55
^3^ Optimum moisture content (%)	22.40	7.90	13.25
^4^ Unified soil classification	MH	SM	CL
^5^ ASSHTO classification	A-7-5	A-1b	A-7-6

^1^ (ASTM D 4318-10), ^2^ (ASTM D 854-10), ^3^ (ASTM D 155710), ^4^ (ASTM D 2487-11), ^5^ (ASTM M 145-91).

**Table 2:** Results of XRF chemical composition of soils.

Composition (%)	Silt	Sand	Clay
SiO_2_	47.9	52.1	51.6
Al_2_O_3_	39.0	19.3	16.9
Fe_2_O_3_	10.7	10.2	13.9
CaO	0.1	8.0	0.36
MgO	0.5	5.1	12.3
Na_2_O	0.1	2.6	-
SO_3_	0.1	-	-
TiO_2_	1.6	1.3	1.17

**Table 3:** Chemical composition of fly ash and lime measured by XRF.

Composition (%)	Fly Ash	Lime
SiO_2_	41.9	1.5
Al_2_O_3_	31.1	1
Fe_2_O_3_	6.4	0.1
CaO	7.4	65.6
MgO	1.5	0.1
Na_2_O	5.6	0.1
SO_3_	1.1	0.3
TiO_2_	1.3	1.1
Loss on ignition—110 °C to 1000 °C	2.1	30.3

**Table 4:** Characteristic size distribution of soils.

Source	D_10_ (μm)	D_50_ (μm)	D_90_ (μm)	Specific Area (m^2^/g)
Silt	3.14	23.45	61.70	0.88
Clay	2.33	16.50	50.24	1.05
Lime	3.85	28.40	106.00	-
Fly ash	12.42	32.94	69.51	0.38

**Table 5:** Compaction test performed.

% mass-soil- stabilizer dosage/moisture (%) - Dry unit weight γd (kN/m^3^)				
100%-silt/ (22.4)-(21.20)	93%-silt +	7% OPC/(23.0)-(16.58)	76%-silt +	24% OPC/(23.4)-(16.80)
		7% L/(23.8)-(15.80)		24% L/(25.8)-(15.24)
		7% FLM/(22.8)-(15.80)		24% FLM/(30.3)-(14.19)
100%-clay/ (13.3)-(19.55)	93%-clay +	7% OPC/(16.0)-(17.92)	76%-clay +	24% OPC/(17.4)-(17.62)
		7% L/(13.7)-(19.55)		24% L/(14.9)-(17.00)
		7% FLM/(14.2)-(17.04)		24% FLM/(14.6)-(16.00)
100%-sand/ (7.9)-(16.22)	93%-sand +	7% OPC/(8.3)-(20.18)	76%-sand +	24% OPC/(8.5)-(18.38)
		7% L/(9.5)-(19.82)		24% L/(12.3)-(18.59)
		7% FLM/(8.3)-(18.38)		24% FLM/(8.5)-(17.31)

## Data Availability

The data are available upon request from the corresponding author.
